# Anterior Chamber Inflammation and Descemet Membrane Endothelial Keratoplasty: An Anterior Segment-OCT-Based Analysis

**DOI:** 10.1016/j.xops.2025.100946

**Published:** 2025-09-23

**Authors:** Sabrina Vaccaro, Giacomo Beschi, Paola Cannistrà, Matteo Airaldi, Marica Ventura, Francesco Semeraro, Vito Romano

**Affiliations:** 1 Eye Unit, Azienda Socio Sanitaria Territoriale (ASST) Spedali Civili di Brescia, Brescia, Italia; 2 Department of Medical and Surgical Specialties, Radiological Sciences, and Public Health, University of Brescia, Brescia, Italy

**Keywords:** Anterior chamber inflammation, AS-OCT, DMEK, Rebubbling

## Abstract

**Purpose:**

To assess anterior chamber (AC) subclinical inflammation using a noninvasive method in patients undergoing Descemet membrane endothelial keratoplasty (DMEK).

**Design:**

Retrospective interventional case series.

**Participants:**

This study included 83 eyes from 73 patients who underwent DMEK surgery and 15 control eyes from 15 healthy individuals.

**Methods:**

The number of hyperreflective dots representing AC cells and optical density ratio (aqueous-to-air relative intensity [ARI] index) for flare quantification were calculated from anterior segment-OCT images. Aqueous-to-air relative intensity index and AC cells were calculated preoperatively and postoperatively at 1 week (T1), 1 month (T2), and 3 months (T3) after DMEK surgery. Baseline values were compared with a healthy control group.

**Main Outcome Measures:**

Anterior chamber cell count and ARI index over time; association with postoperative posterior stromal ripples (PSRs) and rebubbling.

**Results:**

Baseline ARI index was significantly higher in the DMEK group compared with controls, whereas no significant difference in AC cell count was observed. Anterior chamber cell count increased postoperatively from a median of 1.1 cells (0.6–2.1) at baseline to 3.5 (1.7–5.3) at T1 (*P* < 0.001), to 1.7 (1.1–3.0) at T2 (*P* = 0.03), and to 2.1 (1.1–4.2) at T3 (*P* = 0.01). The ARI index also increased from a median of 98.3 (84.1–121.9) at baseline to 142.8 (119.8–221.3) at T1 (*P* < 0.001) and 114.4 (101.7–140.7) at T2 (*P* < 0.001). Higher ARI at T1 was weakly associated with postoperative PSR (odds ratio [OR] = 1.63; 95% confidence interval [CI], 1.00–2.67; *P* = 0.048), whereas postoperative PSR were strongly associated with rebubbling (OR = 26.00; 95% CI, 3.20–211.18; *P* = 0.002).

**Conclusions:**

Anterior segment-OCT enables noninvasive detection of subclinical inflammation after DMEK surgery. The presence of markers of inflammation can increase the risk of early postoperative complications.

**Financial Disclosure(s):**

The authors have no proprietary or commercial interest in any materials discussed in this article.

Descemet membrane endothelial keratoplasty (DMEK) has emerged as a standard therapeutic approach for eyes with endothelial dysfunction, thanks to its faster recovery of visual function.^1^, ^2^, ^3^ Descemet membrane endothelial keratoplasty is a minimally invasive surgical technique, involving the replacement of only the Descemet membrane and endothelium with a significant reduction in surgical trauma.^4^^,^^5^ Nevertheless, DMEK may still induce significant subclinical inflammation.^6^

Surgical trauma and concomitant inflammation disrupt the blood–aqueous barrier, causing the effusion of proteins and inflammatory cells into the anterior chamber (AC).^7^^,^^8^ Studies suggested that inflammation of the aqueous humor is associated with decreased endothelial cell density after corneal transplantation.^9^, ^10^, ^11^ Although levels of several proinflammatory cytokines are significantly reduced after DMEK, their concentrations remain elevated compared with eyes without corneal disease undergoing cataract surgery.^12^

Traditional methods of intraocular assessment such as slit-lamp biomicroscopy have limitations, including poor visibility in cases of corneal edema and high interobserver variability.^13^ Therefore, detecting inflammation with this method is not always possible in patients with ocular edema or scarring. Advances in optical imaging technology offer new opportunities for objective, accurate, and quantitative evaluation of AC inflammation (ACI).^14^

Consequently, some authors have suggested that anterior segment-OCT (AS-OCT), owing to its widespread application in clinical settings, should be employed to measure aqueous inflammation in patients with uveitis.^15^^,^^16^ In this context, the aqueous-to-air relative intensity (ARI) index, derived from AS-OCT images, has been introduced as a noninvasive and reproducible measure of inflammatory activity, complementing slit-lamp biomicroscopy.^15^^,^^16^ However, its role in post-DMEK monitoring remains underexplored. The aim of this study was to assess ACI after DMEK using this imaging-based approach.

## Methods

This retrospective case series included eyes of patients who underwent surgeon prepared DMEK from March 2022 to September 2024 at the ASST Spedali Civili di Brescia. Approval from the local institutional review board was obtained (protocol no. 6210), and the study adhered to the principles of the Declaration of Helsinki.

### Participants and Study Groups

We analyzed all pseudophakic patients with uncomplicated DMEK and for whom preoperative and postoperative AS-OCT data were available. Fifteen pseudophakic eyes from 15 healthy volunteers with intraocular pressure within normal range, without a history of ocular inflammation, injury, surgery in the last 12 months, or significant ocular illnesses were used as a control group. We examined the medical records of all patients receiving DMEK, specifically considering information regarding the best-corrected visual acuity, indication and date of surgery, necessity and date of rebubbling, donor and graft characteristics. A cornea specialist (M.V.) assessed the presence of posterior stromal ripples (PSRs), characterized as an undulation in the posterior corneal profile resembling a ripple, before (preoperative PSR) and after DMEK surgery (postoperative PSR).^17^ Anterior segment-OCT scans were analyzed before surgery (T0) and postoperatively at 1 week (T1), 1 month (T2) and 3 months (T3), where available. Data from AS-OCT were obtained using Casia 2 (Tomey). Anterior segment-OCT scans were conducted during each outpatient clinical visit. Each AS-OCT was analyzed throughout 360° (radial scans), and any detachment was documented. The presence of cystoid macula edema (CME), rejection, and therapy used after the surgery were also recorded.

### Image Acquisition

All AS-OCT scans were acquired with the CASIA2 (Tomey) by a single trained operator, masked to clinical data, under standardized lighting conditions. Patients fixated on an internal target in a darkened room. For each eye, 128 scans were obtained. This standardized protocol was designed to ensure reproducibility and minimize potential measurement bias.

### Image Analysis

#### AC Cells

**Figure 1:**
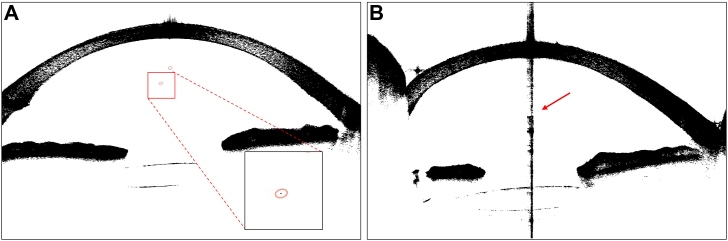
Anterior chamber cells and artifacts. Representative anterior segment-OCT images of artifacts and hyperreflective dots observed in anterior segment imaging. (**A**) Example of an artifact appearing as a strip-like saturation that obscures the corneal surface and blurs the corneal edge (arrow), caused by the incident light source being perpendicular to the tangent of the corneal surface. (**B**) Anterior chamber cells visible as small black dots or points against a white background. Anterior chamber cells are marked by red circles.

Two trained operators, masked to the clinical details of the patient, used a semiautomated method to count the number of distinct hyperreflective dots from 8 evenly distributed OCT images (every 22.5°) of the AC, using Image J (https://imagej.net). The AC was delineated with the polygon selection tool, and images were processed with binarization to reduce background noise. Hyperreflective dots were then manually counted, and the average of the 2 observers’ counts across the 8 scans was recorded as the AC cell count.^16^ Hyperreflective dots can be described as small black dots or points against a white background (Fig 1A). During the scanning process, artifact was defined as a strip-like saturation obscuring the corneal surface and blurring the corneal edge because of the incident light source being perpendicular to the tangent of the corneal surface at that place (Fig 1B).^18^ The average dot count from the 8 cross-sectional images was calculated at each time point.

#### Aqueous Flare

The method employed was described by Invernizzi et al^15^ to estimate aqueous flare. The average brightness value of a 200 × 200-pixel square from the AC area to the right of the apex of the cornea (aqueous intensity) was measured by Adobe Photoshop CS6 (www.adobe.com). The average brightness value of a smaller area of 100 × 100 pixels in the upper right corner of the scan, outside the eye (air intensity) was determined using the same method. The ARI index was defined as the ratio of the brightness value in the AC to that of air. The ARI index values were multiplied by 100 to improve interpretability and scaling, to avoid reporting excessively small decimal values, facilitating clearer comparisons across groups and regression coefficients. The average ARI of 2 images perpendicular to each other and passing through the center was calculated at each time point.

### Surgical Technique

Only cases performed by single senior surgeon (V.R.) were included. The procedure for DMEK surgery was as follows: a circular 9.0-mm descemetorhexis was performed with a reverse Sinskey hook under an air-filled AC. The donor graft was subsequently stained with a 0.06% Trypan blue solution (VisionBlue; DORC International), aspirated into a Geuder injector, and inserted into the recipient AC through a 2.7-mm limbal tunnel incision. All procedures were executed in pseudophakic eyes and without the use of ophthalmic viscosurgical devices. The graft was then fully unfolded over the iris, and an air bubble was injected underneath it to position it onto the recipient’s posterior stroma. The endothelium was oriented toward the recipient’s iris, and the Descemet membrane was oriented toward the recipient’s posterior stroma. Subsequently, the AC was filled with sulfur hexafluoride gas, and an air–liquid exchange was performed, resulting in a gas volume of up to 50%. Subconjunctival dexamethasone was administered after the surgical procedure. Postoperative treatment consisted of antibiotic eye drops and dexamethasone 0.1% drops 6 times daily for 2 weeks. After a steroid regimen consisting of dexamethasone, 0.1% drops were administered 4 times daily for 4 weeks, followed by fluorometholone 0.1% drops administered 4 times daily, which were reduced to once daily at 1 year postoperatively. No systemic corticosteroids were administered. Cases with identified deviations or nonadherence to the therapy were excluded from the analysis.

### Statistical Analysis

The mean ± standard deviation (SD) was used to represent customarily distributed data; otherwise, the median values with interquartile range (IQR) were used.

Parametric and nonparametric tests were selected based on the normality of the data to compare baseline values between DMEK group and control eyes. The D'Agostino and Pearson test and the Shapiro–Wilk test were implemented to evaluate the normality of the data. Mann–Whitney *U* test was used to compare variables when appropriate.

Univariate and multivariate logistic regression analyses were performed to estimate the adjusted odds ratios (ORs) of graft detachment, rebubbling, rejection, diagnosis, preoperative and postoperative PSR, and CME after DMEK. Predictors in the logistic models included the following: AC cells, ARI index, and postoperative PSR.

A mixed-effects linear model was used to estimate the average ARI values, number of cells, central corneal thickness (CCT), and visual acuity at each analyzed time point (T0–T3) for DMEK eyes. The model included time point as a fixed effect and incorporated a random intercept for each eye, along with a random slope for time, to account for intraeye variability. Estimated marginal means were used to obtain the expected values at each time point and to compare estimated means of analyzed variables at each time point.

Generalized additive models were employed to visualize the longitudinal association between time after DMEK surgery and AC cells and ARI value.

Normally distributed variables were analyzed with Pearson’s correlation, whereas nonnormally distributed variables were evaluated with Spearman’s rank correlation. Categorical variables were compared with continuous outcomes using the Mann–Whitney *U* test.

A *P* value <0.05 was considered statistically significant. No imputation of missing data was performed.

Intergrader agreement statistics for cell count and ARI index was assessed using Cohen’s kappa and Bland–Altman analysis on different scans graded independently by 2 masked observers. All graders were masked to clinical data and surgical outcomes to minimize bias.

Prism version 10.4.0 (GraphPad Software Inc), Jamovi version 2.4.1.0 (The Jamovi project), and R version 4.2.2 (R Project for Statistical Computing) were employed to perform the statistical analysis.

## Results

### Demographic and Clinical Data

A total of 93 DMEKs conducted between March 2022 and September 2024 were analyzed. Ten cases were excluded because of incomplete clinical data. Eighty-three eyes from 73 patients who underwent DMEK surgery and 15 control eyes from 15 healthy subjects were included in the analysis. The demographic and clinical features of all participants are summarized in Table 1. No differences were detected between control and DMEK groups with regard to age, sex, intraocular pressure, and CCT distribution (*P* > 0.5).

**Table 1:** Demographic and Clinical Features of DMEK Group

Baseline Characteristic	DMEK Group Patients, *n* = 73; Eyes, *n* = 83
Female sex, *n* (%)	46 (63)
Age, median (IQR), yrs	73.0 (64.50–78.00)
Diagnosis	
FED, *n* (%)	42 (52)
Previous graft failure or others, *n* (%)	16 (18)
PBK, *n* (%)	25 (30)
BCVA, median (IQR), LogMAR	0.50 (0.40–1.30)
IOP, median (IQR), mmHg	14.00 (12.00–15.00)
CCT, median (IQR), μm	696 (620–791)

BCVA = best-corrected visual acuity; CCT = central corneal thickness; FED = Fuchs endothelial dystrophy; IOP = intraocular pressure; IQR = interquartile range; LogMAR = logarithm of the minimum angle of resolution; PBK = pseudophakic bullous keratopathy.

### Quantitative Analysis of AC Cells

**Figure 2:**
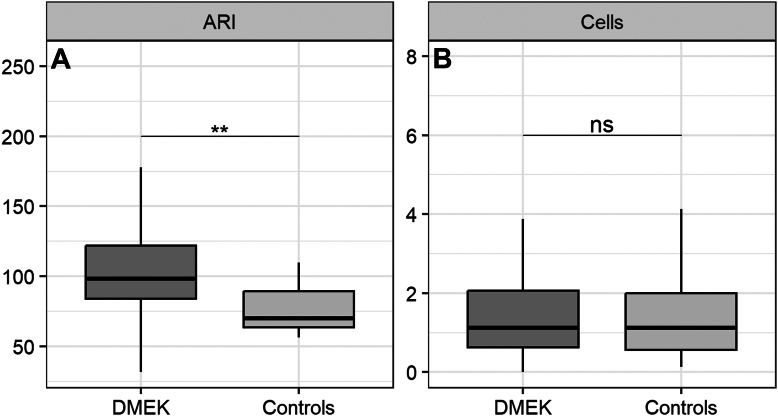
Difference between anterior chamber (AC) cells and aqueous-to-air relative intensity (ARI) values in DMEK and control group at baseline. (**A**) The box plot shows the median number of ARI index for DMEK (*n* = 83) and control group (*n* = 15). There was a significant difference in median ARI index between the 2 groups (Mann–Whitney *U* test, *P* = 0.001). (**B**) No statistical differences were detected in term of AC cells between DMEK and control group (Mann–Whitney *U* test, *P* = 0.87). DMEK = Descemet membrane endothelial keratoplasty; ns = not significant.

**Figure 3:**
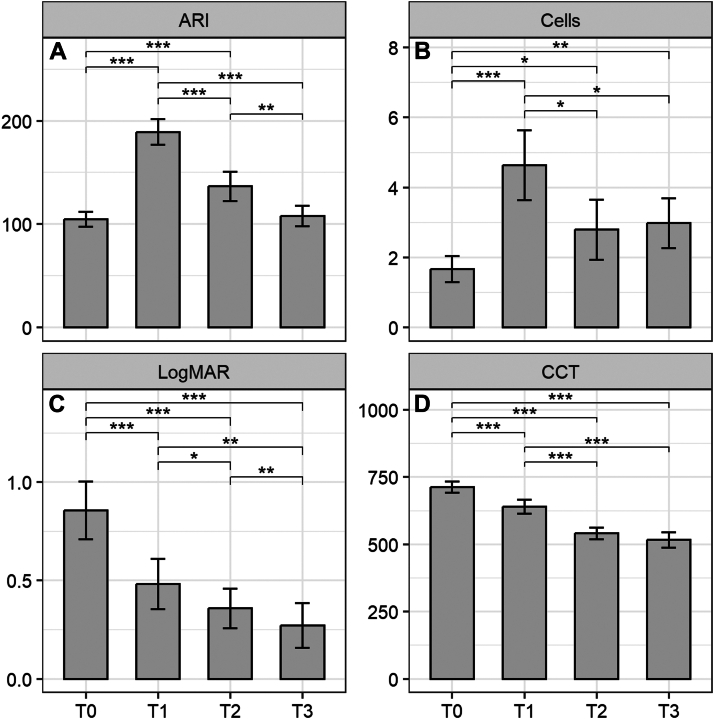
Comparison of estimated aqueous-to-air relative intensity (ARI) index, anterior chamber (AC) cells, central corneal thickness (CCT), and best-corrected visual acuity over time in DMEK eyes. Median values of ARI, AC cells, CCT, and visual acuity expressed in LogMAR at each time point (T0–T3) in DMEK-treated eyes. A mixed-effects linear model was used. (**A**) After DMEK surgery, ARI index significantly increased from baseline to T1 (*P* < 0.001) and T2 (*P* < 0.001). (**B**) Anterior chamber cells increased from baseline to T1 (*P* < 0.001), T2 (*P* = 0.03) and T3 (*P* = 0.01). (**C**) Best-corrected visual acuity improved from baseline to T1 (*P* < 0.001), T2 (*P* < 0.001) and T3 (*P* < 0.001). (**D**) Central corneal thickness decreased from baseline to T1 (*P* < 0.001), T2 (*P* < 0.001) and T3 (*P* < 0.001). DMEK = Descemet membrane endothelial keratoplasty; LogMAR = logarithm of the minimum angle of resolution.

**Figure 4:**
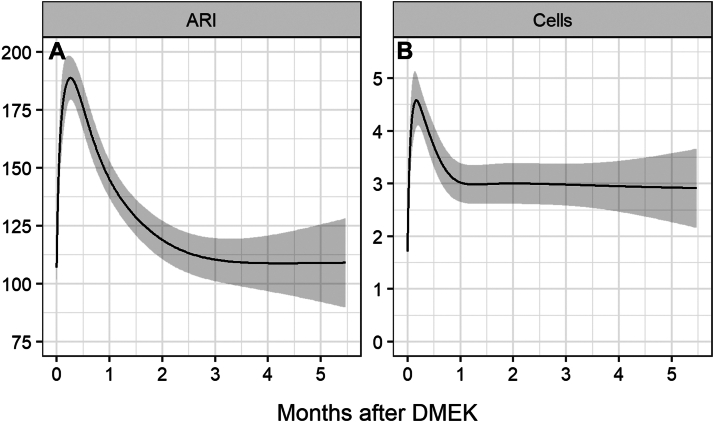
Aqueous-to-air relative intensity (ARI) index and anterior chamber (AC) cells trends after DMEK surgery. Estimated values of ARI (**A**) and AC cells (**B**) after DMEK surgery were derived continuously from longitudinal generalized additive models. DMEK = Descemet membrane endothelial keratoplasty.

No difference was detected between AC cells value of DMEK and control groups at baseline, 1.1 cells (0.6–2.1) versus 1.1 cells (0.6–2.0) respectively (*P* = 0.87) (Fig 2B). The median (IQR) AC cells count increased to 3.5 cells (1.7–5.3) at T1 (*P* < 0.001), to 1.7 cells (1.1–3.0) at T2 (*P* = 0.03), and to 2.1 cells (1.1–4.2) at T3 (*P* = 0.01) (Figure 3, Figure 4B). Anterior chamber cells median (IQR), mean (SD), and estimated means are reported in the supplementary section (Table S1, available at www.ophthalmologyscience.org). The logistic regression analysis did not show any significant association of AC cells count, at both T0 and T1, with graft detachment (OR = 1.16; 95% confidence interval [CI], 0.88–1.52; *P* = 0.28 and OR =1.05, 95% CI [0.94–1.17], *P* = 0.36 respectively), rebubbling (OR = 0.94, 95% CI [0.69–1.30], *P* = 0.73 and OR = 1.03, 95% CI [0.89–1.20], *P* = 0.67 respectively), rejection (OR = 1.13, 95% CI [0.56–2.27], *P* = 0.73 and OR =0.94, 95% CI [0.78–1.14], *P* = 0.55 respectively), preoperative PSRs (OR = 1.08, 95% CI [0.84–1.39], *P* = 0.53 and OR = 1.09, 95% CI [0.97–1.23], *P* = 0.13 respectively), postoperative PSR (OR = 0.99, 95% CI [0.77–1.28], *P* = 0.97 and OR = 1.06, 95% CI [0.94–1.19], *P* = 0.30 respectively) and CME (OR = 1.43, 95% CI [0.65–3.14], *P* = 0.37 and OR = 1.27, 95% CI [0.85–1.89], *P* = 0.23 respectively).

### Quantitative Analysis of Aqueous Flare

A significant difference was detected between median (IQR) ARI values of DMEK and control group at baseline, 98.3 (84.1–121.9) versus 70.0 (63.5–89.3) respectively (*P* = 0.001) (Fig 2A).

The median (IQR) ARI index significantly increased to 142.8 (119.8–221.3) at T1 (*P* < 0.001), to 114.4 (101.7–140.7) at T2 (*P* < 0.001). No statistical differences were detected from the baseline value of 98.3 (84.1–121.9) to 106.6 (94.2–115.6) at T3 (*P* = 0.97) (Figure 3, Figure 4A).

### OR

Aqueous-to-air relative intensity index values at T0 were independently associated with the presence of preoperative PSR (OR = 3.61, 95% CI [1.09–11.94], *P* = 0.03). No statistical significance was detected including CCT and diagnosis (OR = 1.50, 95% CI [0.40–5.06], *P* = 0.54).

In addition, univariate logistic regression analysis did not show any significant association between ARI at T1 and preoperative PSR (OR = 0.82, 95% CI [0.55–1.23], *P* = 0.34).

Univariate logistic regression analysis proved that higher ARI values at T1 were independently associated with the presence of postoperative PSR (OR = 1.63, 95% CI [1.00–2.67], *P* = 0.048). No statistical significance was detected including CCT and diagnosis (OR = 1.36, 95% CI [0.80–2.33], *P* = 0.25).

In addition, logistic regression analysis did not show any significant association between ARI at T0 and postoperative PSR (OR = 0.61, 95% CI [0.21–1.75], *P* = 0.36).

Univariate logistic regression analysis did not show any significant association of ARI values at both T0 and T1 with graft detachment (OR = 0.38, 95% CI [0.12–1.17], *P* = 0.09 and OR =1.23, 95% CI [0.82–1.86], *P* = 0.30 respectively), rejection (OR = 0.70, 95% CI [0.07–6.22], *P* = 0.75 and OR =1.29, 95% CI [0.42–3.91], *P* = 0.65 respectively), CME (OR = 2.00, 95% CI [0.20–19.5], *P* = 0.55 and OR =0.78, 95% CI [0.44–1.40], *P* = 0.41 respectively) and rebubbling (OR = 0.31, 95% CI [0.09–1.10], *P* = 0.07 and OR = 1.48, 95% CI [0.74–2.93], *P* = 0.26 respectively).

### CCT, best-corrected visual acuity, PSR, and Donor/Graft Characteristics

The median (IQR) CCT values were significantly lower after surgery from the baseline value of 696 μm (620–791) to 640 μm (572.5–697.5) at T1 (*P* < 0.001), to 524 μm (496.5–553) at T2 (*P* < 0.001) and to 517 μm (483–537) at T3 (*P* < 0.001) respectively (Fig 3D). There was an improvement in best-corrected visual acuity as well, from the median (IQR) baseline value of 0.5 logarithm of the minimum angle of resolution (LogMAR) (0.4–1.3) to 0.3 LogMAR (0.1–0.5) at T1 (*P* < 0.001), to 0.2 LogMAR (0.1–0.4) at T2 (*P* < 0.001) and to 0.04 LogMAR (0.03–0.3) at T3 (*P* < 0.001) respectively (Fig 3C). Postoperative PSR was associated with rebubbling occurrence (OR = 26.00, 95% CI [3.20–211.18], *P* = 0.002). In addition, no significant correlations were found between donor or graft characteristics (donor age, sex, endothelial cell density, graft diameter) and ARI or cell values at T0, T1, and T2.

### Intergrader Reliability

Intergrader agreement statistics for cell count and ARI index Cohen’s κ ranged from 0.94 and 0.99 (Table S2, available at www.ophthalmologyscience.org). Bland–Altman analysis demonstrated a mean difference in measurements of 0.59 units between the 2 operators for ARI values, with limits of agreement ranging from –30.94 to 32.12 (mean ± 1.96 SD). The mean difference for cell values was 0.02 units, with limits of agreement ranging from –0.11 to 0.14 (mean ± 1.96 SD) (Fig S1, available at www.ophthalmologyscience.org).

## Discussion

In the present study, we quantified ACI in eyes that underwent DMEK surgery using a noninvasive method, and we found that both the ARI index and AC cells increased after surgery before returning to baseline values. Higher inflammation levels were associated with the presence of postoperative PSRs, an independent marker of rebubbling risk.

DMEK, although less invasive than other keratoplasties, may nonetheless cause subclinical inflammation.^6^ Slit-lamp evaluation of ACI is common but limited in cases of corneal opacity or edema.^14^^,^^19^ Anterior segment-OCT offers a noninvasive, high-resolution alternative for anterior segment imaging and has become a standard tool in corneal transplant follow-up.^20^ To assess ACI, we examined estimated aqueous flare and AC cells before and after DMEK surgery. We examined these values within the first postoperative months to further assess the re-establishment of the blood–aqueous barrier after surgery.^12^ Both parameters increased postoperatively, peaked during the first week, and gradually declined over time yet remained above preoperative levels.

The baseline estimated aqueous flare exhibited substantial variation between the control group and the DMEK group. In line with our findings, a previous study demonstrated that eyes undergoing DMEK showed elevated levels of the proinflammatory mediators interferon-γ, interleukin-8, and interleukin-10 compared with eyes without corneal disease scheduled for cataract surgery.^12^ On the contrary, the number of AC cells was not statistically different between the 2 groups. This can be explained by the fact that most AC cells represent inflammatory cells, which appear as hyperreflective dots on AS-OCT, as described by Agarwal et al.^21^ Probably, proinflammatory mediators are likely not discernible as hyperreflective spots and induce chronic subclinical inflammation. Therefore, it may be crucial to evaluate the inflammatory condition, which can manifest as a clinically undetectable immune response.

Interestingly, despite a significant increase in ACI postoperatively, neither AC cell counts, nor estimated aqueous flare values were significantly associated with graft detachment, rejection, or CME. This could be attributed to the low incidence of postoperative complications in our cohort, potentially underpowering these analyses. The objective quantification of pre and postoperative inflammation in the AC is crucial to assess the correlation between inflammation levels and the risks of detachment, rebubbling and rejection. Slit-lamp examination is not objective and can be operator dependent. Prior studies have demonstrated poor interobserver agreement in the evaluation of ACI, particularly concerning flare grading.^22^ Alternative techniques, such as the laser flare photometer, have been employed to evaluate AC activity. Laser flare photometry offers reliable and precise measures of AC flare and cells, although it is hardly utilized in standard clinical practice because of the absence of consensus and its high cost.^23^ Consequently, some authors have proposed that AS-OCT, owing to its extensive availability in clinical practice, may be utilized to evaluate flare and cells in the AC, particularly in the case of uveitis.^21^^,^^24^ In this regard, Invernizzi et al^15^ introduced a standardized approach for quantifying inflammation in the AC using AS-OCT and compared it with findings from laser flare photometer analysis and clinical cell grading of the same uveitis patients. A positive correlation was identified between OCT-derived AC inflammatory indices and conventional clinical indicators.

We choose to examine the subgroups according to both preoperative and postoperative factors that have been documented to affect postoperative outcomes in DMEK surgery to clarify the potential correlations between noninvasively measured imaging results via AS-OCT and clinical outcomes. In the literature, numerous preoperative and postoperative variables that lead to graft detachment and rebubbling after DMEK surgery have been identified.^25^, ^26^, ^27^, ^28^ We found that ARI values at T1 were independently associated with the presence of postoperative PSR. The presence of postoperative PSR has consistently been associated with an increased risk of rebubbling, a finding that has been confirmed by the present study.^17^^,^^29^ In particular, Coco et al^17^ reported that the risk of graft detachment requiring rebubbling at any time was substantially correlated with the presence of stromal ripples. Ventura et al^29^ also confirmed that postoperative PSR confer a higher risk of rebubbling. Although PSR require multicenter scale validation, our findings suggest that it may provide practical insights into postoperative graft behavior. Posterior stromal ripples may represent an advanced manifestation of corneal edema, occurring when swelling extends throughout the posterior stroma.^30^

Various studies have investigated the impact of ACI on corneal endothelium. Some research suggests that the presence of ACI correlates with significantly decreased endothelial cell density in comparison to healthy controls.^31^ Several potential factors relate ACI to endothelial injury, including direct harm from proteins and inflammatory cells present in the aqueous humor.^31^ In the present study we quantified ACI in eyes that underwent DMEK surgery using a noninvasive method. Anterior segment-OCT offers the potential to detect postoperative inflammatory reactions which may subsequently cause endothelium damage, with easy to obtain acquisition, in vivo and noninvasively. At the same time this finding could facilitate clinical decision-making by offering real-time, detailed imaging that supports early detection and monitoring of potential complications, allowing treatments and more personalized patient care.

This study has several limitations. Because of its retrospective design, there was no control over the distribution of preoperative characteristics among DMEK patients, and selection bias cannot be fully excluded. The exclusion of cases with incomplete follow-up may have led to an underrepresentation of eyes with prolonged inflammation or late complications. This study included a small subset of patient with both eyes undergoing DMEK. To account for potential intrapatient correlation of outcomes we employed linear mixed-effects models, which appropriately adjust for the non-independence of observations within the same patient. Although this approach reduces the risk of statistical bias, we recognize that residual correlation between fellow eyes may remain. Another important limitation of this study is the relatively small number of patients in certain subgroups, especially the low number of eyes with previous graft failure. Therefore, while no statistical differences were observed among preoperative diagnoses, these findings should be interpreted with caution because of the limited sample size. To minimize operator bias, AS-OCT image analyses were conducted independently by 2 trained examiners masked to clinical data. Although some overlap may occur between hyperreflective features, classifications were based on spatial location and morphology. We acknowledge the potential for interpretative ambiguity and recommend future validation studies to standardize these descriptors. Other possible limitations of AS-OCT imaging are the difficulty in distinguishing inflammatory cell types, the image quality variability, and potential artifacts because of interface gas or movement. Furthermore, this technique is difficult to use in the first postoperative day because residual gas in the AC makes measurements of intracameral inflammation imprecise and patient discomfort limits acquisition. Moreover, although this quantitative AS-OCT-based approach offers a promising tool for evaluating ACI after DMEK, larger-scale prospective studies are necessary to validate its clinical applicability. Specifically, we did not identify any relationship between rejection, detachment, rebubbling or CME with either the ARI score or AC cells. The lack of such a relationship may be explained by the low incidence of postoperative complications in the included population. Finally, prospective studies are essential to determine a cut-off that can be useful for a more comprehensive interpretation of the imaging results and their potential application in postoperative therapy management. In particular, patients with elevated estimated aqueous flare may benefit from tailored corticosteroid regimens aimed at reducing subclinical inflammation in the AC.

In conclusion, our study suggest that AS-OCT could serve as a valuable adjunct in post-DMEK monitoring, particularly for detecting subtle inflammatory responses that may influence graft adhesion. Its systematic use in the postoperative setting could help refine patient stratification, identify eyes at higher risk of complications, and potentially guide earlier therapeutic interventions. In the future, integrating quantitative indices of inflammation obtained with AS-OCT into routine care may inform personalized treatment strategies aimed at improving long-term graft survival. Prospective studies are needed to confirm whether AS-OCT guided management can translate into better clinical outcomes.
