# Supplementary material for: Anterior Chamber Inflammation and Descemet Membrane Endothelial Keratoplasty: An Anterior Segment-OCT-Based Analysis
Source: Ophthalmol Sci. 2025 Sep 23;6(1):100946. doi: 10.1016/j.xops.2025.100946 (PMC12613106; doi:10.1016/j.xops.2025.100946)
Supplement: Supplementary Table 1 [file mmc1.pdf]

Supplementary Table 1: Intraclass Correlation Coefficient (ICC) for aqueous-to-air relative intensity (ARI) index and anterior chamber cell counts (cells)

	**Variable**	**Time**	**ICC**	**Lower**	**Upper**
**1**	ARI	T0	0.949	0.923	0.967
**2**	ARI	T1	0.970	0.954	0.981
**3**	ARI	T2	0.999	0.999	0.999
**4**	ARI	T3	0.999	0.999	0.999
**5**	Cells	T0	0.999	0.999	0.999
**6**	Cells	T1	0.999	0.999	0.999
**7**	Cells	T2	0.999	0.999	0.999
**8**	Cells	T3	0.999	0.999	0.999
