# Supplementary material for: Anterior Chamber Inflammation and Descemet Membrane Endothelial Keratoplasty: An Anterior Segment-OCT-Based Analysis
Source: Ophthalmol Sci. 2025 Sep 23;6(1):100946. doi: 10.1016/j.xops.2025.100946 (PMC12613106; doi:10.1016/j.xops.2025.100946)
Supplement: Supplementary Table 2 [file mmc2.pdf]

Supplementary Table 2: Aqueous-to-air relative intensity (ARI) index, central corneal thickness (CCT), anterior chamber (AC) cell counts (cells) and best correct visual acuity (BCVA) expressed in LogMAR at each postoperative time point. Median and interquartile range (IQR), mean and standard deviation (SD), and estimated means from the linear mixed-effects model are reported.

Variable	Time	Eyes	Median_IQR	Mean_SD	EstimateMean_95_CI	Comparison p values		
						T1	T2	T3
ARI	T0	83	98.3 (84.1, 121.9)	104.8 (40.9)	104.7 (94.1, 115.4)	< 0.001	< 0.001	0.97
ARI	T1	78	142.8 (119.8, 221.3)	188.82 (111.0)	188.9 (164.3, 213.4)	-	< 0.001	< 0.001
ARI	T2	74	114.4 (101.7, 140.7)	136.6 (62.9)	136.6 (125.3, 147.9)	-	-	0.01
ARI	T3	55	106.6 (94.2, 115.6)	108.6 (31.46)	108.4 (95.3, 121.6)	-	-	-
CCT	T0	81	696 (620, 791)	712.4 (122.32)	712.2 (691.6, 732.7)	< 0.001	< 0.001	< 0.001
CCT	T1	76	640 (572.5, 697.5)	640.43 (112.2)	639.9 (613.8, 666)	-	< 0.001	< 0.001
CCT	T2	75	524 (496.5, 553)	541.92 (85.15)	540.7 (519.2, 562.1)	-	-	0.51
CCT	T3	57	517 (483, 537)	517.46 (56.79)	516.3 (488.1, 544.5)	-	-	-
Cells	T0	83	1.1 (0.6, 2.1)	1.7 (1.7)	1.7 (1.3, 2)	< 0.001	0.03	0.01
Cells	T1	78	3.5 (1.7, 5.3)	4.6 (4.5)	4.6 (3.6, 5.6)	-	0.04	0.03
Cells	T2	74	1.7 (1.1, 3.0)	2.7 (3.8)	2.8 (1.9, 3.6)	-	-	0.99
Cells	T3	55	2.1 (1.1, 4.2)	3.0 (2.5)	3 (2.3, 3.7)	-	-	-
LogMAR	T0	82	0.5 (0.4, 1.3)	0.86 (0.68)	0.9 (0.7, 1)	< 0.001	< 0.001	< 0.001
LogMAR	T1	50	0.3 (0.1, 0.5)	0.45 (0.58)	0.5 (0.4, 0.6)	-	0.03	2
LogMAR	T2	69	0.2 (0.1, 0.4)	0.34 (0.44)	0.4 (0.3, 0.5)	-	-	0.01

LogMAR	T3	52	0.04 (0.03, 0.3)	0.24 (0.45)	0.3 (0.2, 0.4)	-	-	-
