# Supplementary figures and images for: Anterior Chamber Inflammation and Descemet Membrane Endothelial Keratoplasty: An Anterior Segment-OCT-Based Analysis
Source: Ophthalmol Sci. 2025 Sep 23;6(1):100946. doi: 10.1016/j.xops.2025.100946 (PMC12613106; doi:10.1016/j.xops.2025.100946)

Bland-Altman plot of interobserver agreement

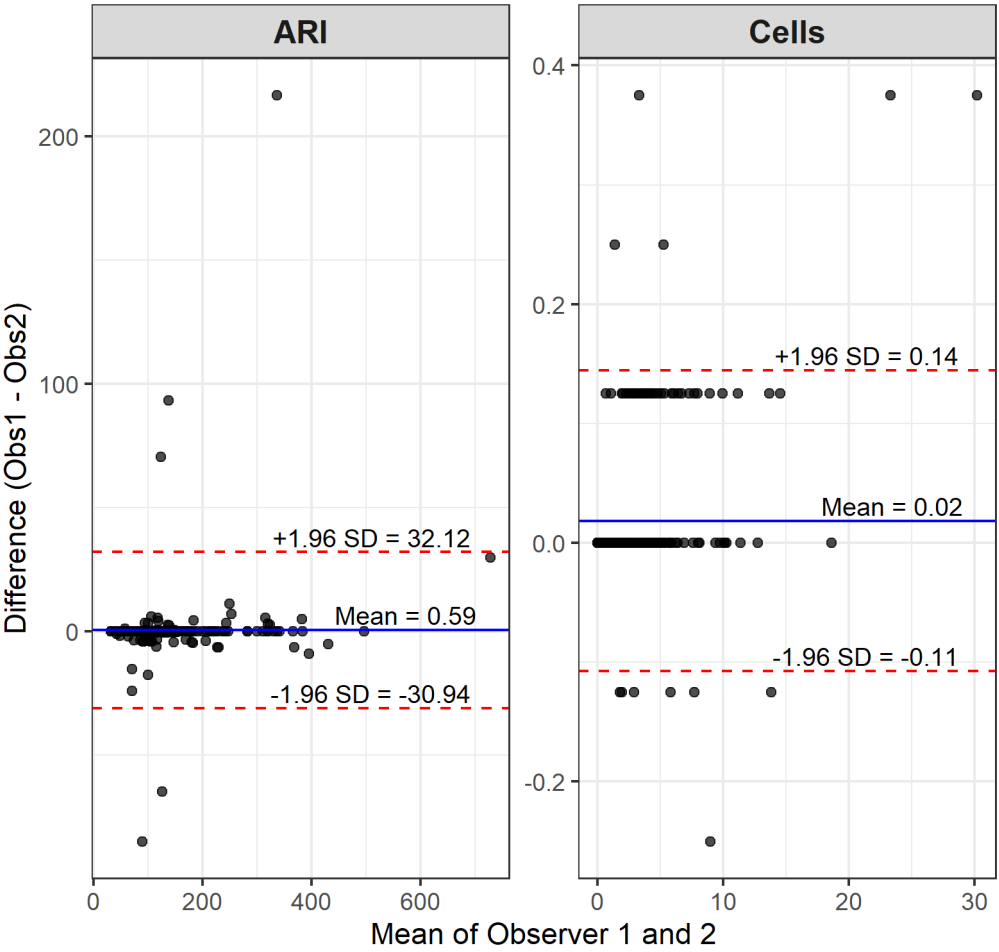

Supplement: Supplementary figure 1 [file mmc3.pdf]
